# Predicting the COVID-19 Pandemic: The Perceptions of Healthcare Workers and the General Public

**DOI:** 10.7759/cureus.12615

**Published:** 2021-01-11

**Authors:** Francine Cheese, Harry Coulton

**Affiliations:** 1 Cardiology, Bristol Royal Infirmary, Bristol, GBR; 2 Internal Medicine, Southmead Hospital, Bristol, GBR

**Keywords:** covid-19, novel coronavirus, coronavirus disease 2019, covid-19 pandemic, covid-19 forecasting, forecasting, public health and safety, knowledge on covid-19, healthcare provider, covid-19 uk

## Abstract

Background: Coronavirus Disease 2019 (COVID-19) is a pandemic of significant international concern, requiring decisive government actions with public understanding and subsequent adherence to control the spread. This study investigated the predictions of the public and healthcare workers (HCWs) at an early stage of the United Kingdom (UK) pandemic to assess their understanding of this novel virus and provide a reflection of the information readily available to them at the time.

Method: A cross-sectional survey between the 18th and 20th March 2020 of UK adults was conducted via an anonymous 17-question online questionnaire using a snowball sampling technique. Simple descriptive statistics, repeated measures analysis of variance (ANOVA), and unpaired Mann-Whitney t-tests investigated significance at the P<·05 levels.

Results: A total of 823 UK residents responded, of which 12·0% (n=99) were HCWs (doctors and nurses). The primary information sources used by our participants were BBC News, group messaging such as WhatsApp, and NHS England. The majority (38·9%) estimated government-enacted social restrictions would last two to four weeks. Mean best guess of total UK COVID-19 mortality was 1000 to 10,000 deaths, and the majority of participants (77·9%) revealed that they expected their day-to-day lives to be affected for less than six months in total. HCWs consistently estimated greater duration, scale, and impact of COVID-19 than non-healthcare workers (Non-HCWs).

Conclusion: Survey respondents greatly underestimated the duration and impact of COVID-19 on their personal and public lives. Non-HCWs made greater underestimates than HCWs. This provides a historical reference and highlights a lack of clear information regarding the pandemic at the time of the survey. There is an ongoing need for effective, realistic, and timely communication between government, front-line clinicians, and the general public to manage expectations of the course of the pandemic and, consequently, increase adherence to public health measures.

## Introduction

Following the identification of severe acute respiratory syndrome coronavirus 2 (SARS-CoV-2), which causes Coronavirus Disease 2019 (COVID-19), in December 2019 in China, rapid worldwide spread led the World Health Organisation (WHO) to declare COVID-19 an emergency of international concern [1]. In the United Kingdom (UK), community transmission was identified in March 2020, and restrictive public health measures were thereafter implemented in response to official forecasting data predicting large numbers of deaths as a result of an overwhelmed health service [2].

During this early phase of the pandemic in the UK, it was essential for the general public, clinicians, and the government to stay up-to-date with the latest information in order to make informed choices in their private and public lives. However, in an era with freely available mass information, which is frequently unverified and possibly inaccurate, it can be a difficult task to navigate and identify facts from fiction. Often, front-line clinicians found themselves acting as the trusted information source for friends, family, and the wider public - a role with great responsibility [3-4].

Misconceptions surrounding a pandemic have been shown to result in a lack of protective behaviours [5] and reduced adherence to public health guidelines [6-7]. Compliance is particularly poor if public health measures last for longer than expected by the individual [8]. Poor compliance and risky behaviours lead to wider viral spread and increased deaths. It is, therefore, a vital responsibility of the government to provide realistic and accessible data regarding COVID-19, which clinicians and the general public can use to form their own accurate expectations of the course of the pandemic. 

To investigate the government’s success in providing accessible and understandable information, we asked healthcare workers (HCWs) and non-healthcare workers (Non-HCWs) to forecast the course of the pandemic. These predictions were collected in March 2020, prior to the national ‘lockdown’ whereby citizens had to stay at home other than for daily exercise or essential tasks. The results were then compared to the official forecasts as well as real outcomes in November 2020. The accuracy of these predictions provides a historical snapshot of the accessibility of data available to HCWs and Non-HCWs at the time, and their subsequent knowledge and understanding of the likely course COVID-19 would have on the UK.

This article was previously presented as a meeting abstract at the 2020 MedAll International Virtual Medical Conference (IVMC): Navigating Uncertain Waters on May 23rd, 2020.

## Materials and methods

Survey circulation and participants  

A cross-sectional survey was conducted via an anonymous 17-question online questionnaire, disseminated to UK adults using a snowball sampling technique using WhatsApp and email. Participation was voluntary and anonymous and without monetary compensation. Informed consent was gained for the collection of data and its use in publication.

The survey opened on 18/03/20. Responses between 12:00 (GMT) on the 18/03/20 and 16:00 (GMT) on the 20/03/20 were interpreted prior to the announcement of a UK national 'lockdown'. The survey was a structured questionnaire with single and multiple-choice answers. Participants were asked a series of questions, including sociodemographics, forecast predictions of duration, impact, and UK mortality from COVID-19. Participants were asked to state their profession, allowing comparison between HCWs and Non-HCWs. See Appendix 1 for the full survey.  

Analysis   

Analyses were performed using Prism 8 (GraphPad Software Inc, La Jolla, California, US). Simple descriptive statistics, repeated measures analysis of variance (ANOVA), and unpaired Mann-Whitney t-tests were used. Results were considered statistically significant at P<·05.   

## Results

This study captured the UK HCWs' and Non-HCWs' perception of COVID-19 in the context of a rapidly evolving pandemic. A total of 823 responses were collected from all UK locations. The majority of respondents were from England (92·5%), followed by Scotland (3·8%), Wales (2·7%), and Northern Ireland (1·0%). Twelve percent were HCWs (n=99). Participants were predominantly younger than 39 years (72·9%). Age demographics can be found in Table 1.  

**Table 1:** Total Number and Percentage of Survey Respondents by Age (Years)

Age Group of Respondents	Number of Respondents
18-24	206 (25·0%)
25-39	394 (47·9%)
40-49	75 (9·1%)
50-59	89 (10·8%)
60-69	44 (5·3%)
70+	15 (1.8%)

Information sources

**Figure 1:**
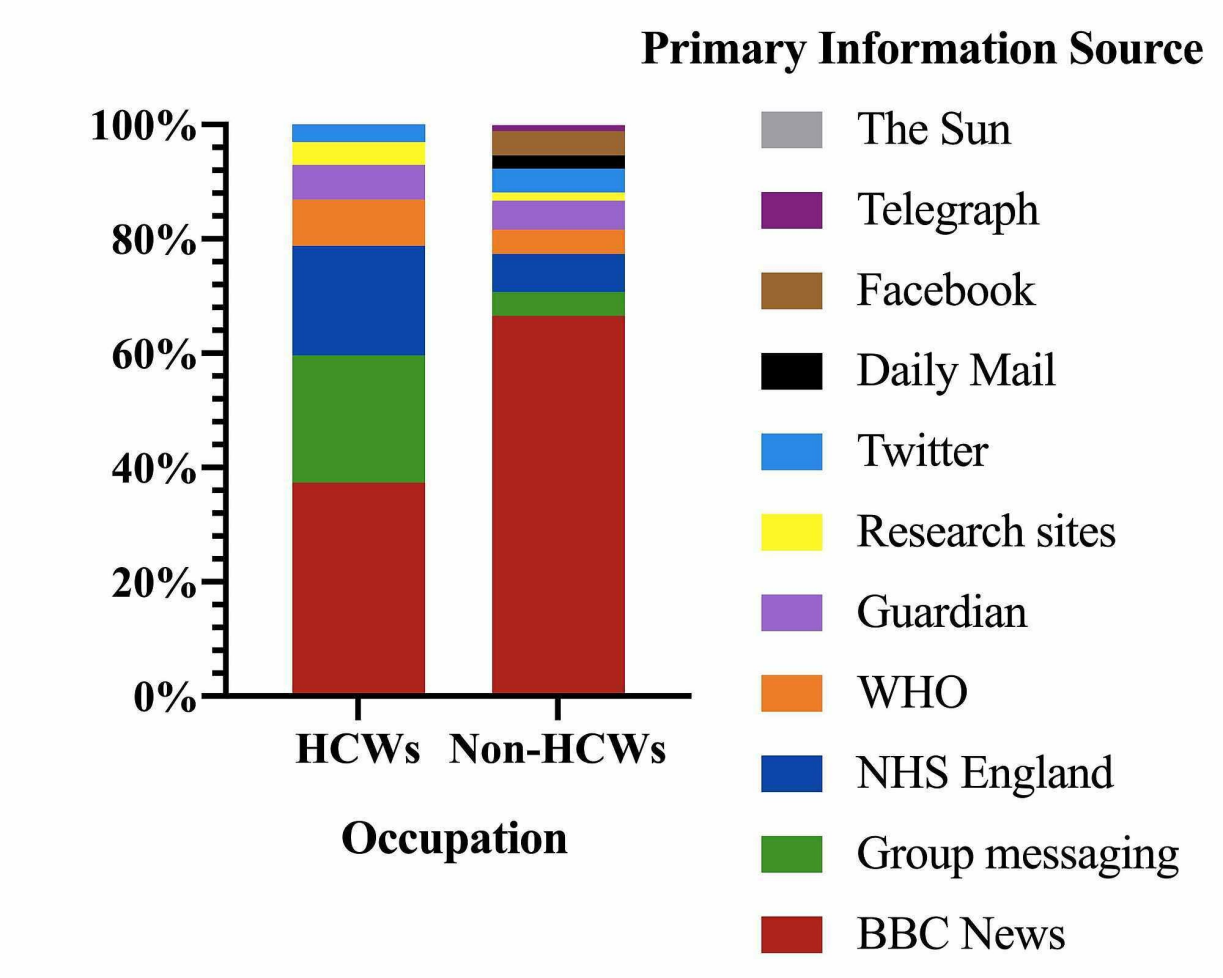
Primary Information Sources Compared by Occupation

The majority of HCWs and Non-HCWs used BBC News, group messaging services such as WhatsApp, and NHS England as their primary information sources. HCWs reported less use of BBC News than Non-HCWs, at 37·4% compared to 66·6%. However, they reported significantly more group messaging at 22·2% to 4·1% (Figure 1).

Public health measures

One hour after the closure of our survey, the UK government announced plans for a national lockdown, which was consequently implemented three days later, on the 23rd of March. Of our respondents, 9·8% reported that there would be no UK lockdown, with 18·3% predicting that such restrictions would occur, but lasting less than two weeks, and 38·5% expecting between two and four weeks. No respondents (0%) forecasted lockdown greater than 12 months (Table 2). There was no significant difference in response by age groups (data not shown). HCWs, on average, estimated longer duration of restrictions than Non-HCWs (P<·05, unpaired Mann-Whitney t-test).

**Table 2:** Forecasted Duration of a UK Public Lockdown by HCWs and Non-HCWs Expected durations of public lock down, as reported by HCWs (Doctors and Nurses, n=99) and Non-HCWs (n=728); * indicates median response. HCWs: 25% percentile = 2-4 weeks, 75% percentile = 1-2 months. Non-HCW: 25% percentile = 1-2 weeks, 75% percentile = 1-2 months

	No Lockdown	1-2 Weeks	2-4 Weeks	1-2 Months	2-4 Months	4-12 Months	Greater than 12 Months
HCWs	10·1% (10)	12·1% (12)	35·4% (35)*	23·2% (23)	16·1% (16)	3% (3)	0% (0)
Non-HCWs	9·8% (71)	19·2% (139)	39·0% (282)*	22·9% (166)	8·0% (58)	1.1% (8)	0% (0)

Duration of impact

When asked to forecast how long COVID-19 would impact on day-to-day life, of all respondents, the majority predicted three to four months in total (35·7%). Overall, 77·9% predicted an impact for less than six months. There was no significant difference in response by age groups (data not shown).

Median prediction of impact duration by HCWs were greater than Non-HCWs (Table 3), as was the mean response at P<·0001 level (unpaired Mann-Whitney t-test).

**Table 3:** Forecasted Duration of COVID-19 Impacting Day-to-day Life by HCWs and Non-HCWs Expected durations of COVID-19 pandemic impact on day-to-day life, as reported by HCWs (Doctors and Nurses, n=99) and Non-HCWs (n=728); * indicates median response. HCWs: 25% percentile = 3-4 months, 75% percentile = 6-12 months. Non-HCWs: 25% percentile = 3-4 months, 75% percentile = 4-6 months.

	Less than 1 month	1-2 months	3-4 months	4-6 months	6-12 months	Over 12 months
HCWs	0% (0)	8·1% (8)	24·2% (24)	27·3% (27)*	24·2% (24)	16·2% (16)
Non-HCWs	2·2% (16)	22·4% (162)	37·3% (270)*	18·5% (134)	12·0% (87)	7·6% (55)

Mortality estimates  

**Figure 2:**
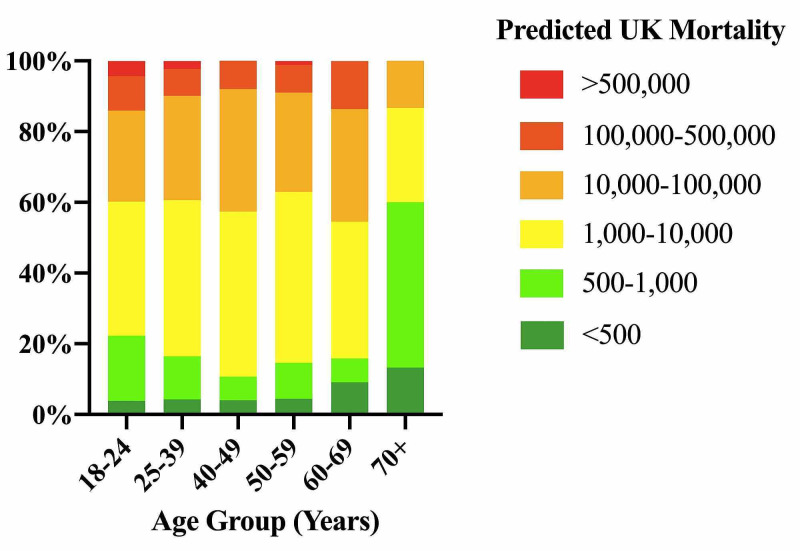
Forecast Estimates of Number of UK Deaths as a Result of the COVID-19 Pandemic, by Age Y-axis represents cumulative percentage (%) response.

The majority of respondents (42·6%) predicted that the UK would have in total between 1000 and 10,000 deaths. Few forecasted over 500,000 deaths (2·3%) or under 500 deaths (4·6%). Mean response of the 70+ age group was significantly lower than that of any other age group, at 500 to 1000 UK deaths (P<·05, Kruskal-Wallis ANOVA with Dunn’s multiple comparison test, n=15-210/age group) (Figure 2).

The mean response of HCWs was significantly higher than that of Non-HCWs, at the P<·05 level (unpaired Mann-Whitney t-test) (Table 4).

**Table 4:** Forecasting of Total UK Mortality by HCWs and Non-HCWs HCWs and Non-HCWs forecast estimates of the number of UK deaths as a result of the COVID-19 pandemic; * indicates median response. HCWs: 25% percentile = 1k-10k, 75% percentile = 10k-100k. Non-HCWs: 25% percentile = 1k-10k, 75% percentile = 10k-100k

	< 500	500 – 1,000	1,000 - 10,000	10,000 - 100,000	100,000 - 500,000	> 500,000
HCWs	3% (3)	7·1% (7)	42·4% (42)*	37·4% (37)	9·1% (9)	1% (1)
Non-HCWs	4·8% (35)	14·2% (103)	42·7% (309)*	27·3% (198)	8·4% (61)	2·5% (18)

## Discussion

We asked our respondents to forecast the probable duration and impact of the COVID-19 pandemic in the UK at a critical time, ahead of the announcements regarding a national lockdown and prior to the commencement of governmental officials on national television giving daily briefings regarding COVID-19. Although it is recognised that predicting the course of a pandemic is difficult, even by March 2020, it was clear that COVID-19 would have a far greater impact than suggested by our respondents.

On March 20th, 2020, at 17:00, the UK Prime Minister delivered a speech on national television outlining a lockdown [9]. These restrictions to hospitality, as well as venues such as beauticians, hairdressers, and gyms, continued for the next two and a half months, with a second mandatory closure implemented on November 5th, 2020. However, just hours previously, nearly 1 in 10 of our respondents (9·8%) had stated that there would never be a lockdown in the UK, with a further 56·9% of respondents expecting a lockdown to occur, but with restrictions to last a maximum of four weeks. These clear underestimations were also seen in our respondent’s forecasting of the duration that COVID-19 would affect their day-to-day lives, with 77·9% estimating an impact of fewer than six months in total. This reveals a significant gap between the expectations and understanding of the public compared to government officials at that time, who were modelling their COVID-19 response on data released on the 16th March, which suggested transmission would not be fully controlled for 18 months or more [2].

This gap in understanding was also revealed when comparing mortality estimates. Over the time our survey was open, UK deaths rose from 53 to 82 [10]. Predictions of mortality estimates at the time from the joint University College London and University of Cambridge and Health Data Research suggested that the UK would have a total of between 35,000 and 70,000 deaths [11], whilst a report by Imperial College London predicted nearer 250,000 with mitigation measures [2]. In reality, as of November 5th 2020, the UK has reported in excess of 47,000 COVID-19 attributed deaths [12]. In contrast, our participants aged 18-69 estimated UK mortality at a total of 1000 to 10,000, with 60·6% of all respondents predicting a mortality total of less than 10,000. The more elderly respondents (70+ age group) were even poorer predictors, estimating 500 to 1000 deaths.

It is known that accurate forecasting relies on two key skills: The amalgamation of evidence and the understanding of that evidence to create a realistic prediction [13]. Therefore, the fact that our respondents gave large underestimates of impact, duration and mortality, despite the official forecasting data being publicly available, suggests that at the time of the survey, there were barriers to the public accessing or understanding this information about COVID-19. This is concerning. Underestimation of personal risk correlates with that individual exhibiting riskier behaviour [5] and public health measures exceeding the expected duration are less likely to be adhered to [8]. This has been evidenced in recent compliance, with as low as 10·9% self-isolating when directed to do so by their NHS app [14], despite a survey at the start of the pandemic reporting that 88% would self-isolate if required [15]. These behaviours likely lead to increased spread of the pandemic.

When considering the reasons for the lack of knowledge, one key variable to consider is the information sources relied on by the public. Multiple studies have found WhatsApp and social media to be key information sources utilised in this pandemic, with citizens relying on them in preference to official public health websites [16-17]. However, social media has been known to spread false information [18], and lead to an underestimation of the severity of COVID-19 as compared to those who use government websites [19-20]. Our study supported the literature but suggested that although group messaging services such as WhatsApp were used by a significant proportion of our respondents (22·2% of HCWs and 4·1% of Non-HCWs), the majority used BBC News as their primary information source (37·4% of HCWs and 66·6%. Of Non-HCWs). This highlights the responsibility of the BBC during this pandemic to deliver accurate and current information.

An encouraging finding from our survey was that HCWs predicted a longer duration of impact on day-to-day life, duration of lockdown and overall mortality, suggesting a greater overall understanding of COVID-19. This is supported by other studies, who found that knowledge of the pandemic is significantly associated with profession and educational level [21], and overall knowledge regarding COVID-19 is high in HCWs [15,22]. Furthermore, HCWs have greater access to expert colleagues’ opinions, as well as more experience of tackling infectious diseases, perhaps recognising that COVID-19 would remain an issue until a widespread vaccination program or effective therapies were available. Interestingly, our study revealed a far greater reliance on group messaging services (i.e. WhatsApp) by HCWs than Non-HCWs, at 22·2% compared to 4·1%. In this case, although our survey did not investigate the content of information communicated through group messaging, we postulate that it was used by HCWs to share relevant COVID-19 updates.

However, it must be noted that HCWs predictions were still hugely inaccurate. This is concerning given that HCWs were relied upon to become both formal and informal information sources for the general public [18,23], with great trust placed in HCWs to provide accurate information. This trust is diluted when an individual HCW has a poor understanding of the pandemic [24]. Therefore, if HCWs trivialised the possible impact of the pandemic, this could have far-reaching consequences on the public’s risk behaviours, as well as overall trust in the profession. This highlights the importance of government officials providing a unified accessible source of information for HCWs and Non-HCWs alike. This has been recognised by the WHO as a priority [25], whereby communicating trustworthy, clear, and realistic forecasts will enable informed decision making and behaviours. There is also public support for honest and accurate information during epidemics, even if that information is anxiety-provoking [26]. To ensure that this information is far-reaching, government and public health officials should utilise social media, infographics, and other forms of popular communication to their advantage [17,20,21,27] as well as ensuring the elderly generations are included in information campaigns.

The above findings need to be taken into consideration with the timing of our survey and its limitations. This data was collected prior to the UK government initiating daily communication briefings on national television. Consequently, it would be interesting to have compared public knowledge and subsequent forecasting accuracy after these briefings had started, to assess the success of this method of communication. In addition, due to the need for rapid circulation and avoidance of social contact, a snowball strategy was used to gather respondents. This resulted in over-representation of the younger generation, with the interpretation of data from the over 70 years population limited due to sample size. Also, the education level of our respondents was not gathered, which is likely an important variable in an individual’s forecasting skills. Consequently, our data should be interpreted cautiously and is not generalisable to the UK population, with recommendations for future studies to explore forecasting of the more elderly generations and gather further demographic information.

## Conclusions

Forecasting requires an individual to understand and utilise information to predict a realistic probability of an outcome. The more well-informed an individual, the more accurate is the forecast. Our study revealed that in March 2020, the population surveyed displayed an unrealistic underestimate of the scale, impact, and duration of the pandemic. HCWs were better predictors than Non-HCWs but still provided inaccurate estimates compared to the official forecasters and later reality. Overall, this poor forecasting may reflect a lack of clear and detailed communication by government officials. This is of utmost importance: underestimates of risk at an individual level result in more risky behaviour and less adherence to public health guidelines.

This study provides a valuable insight into the public perception of COVID-19 during the crucial initial phases of the pandemic in the UK, and evidences that social media surveys can be a useful tool to monitor public knowledge in a rapidly changing situation. The government has a duty to act on such findings to manage the expectations of the public and provide clear communication in an accessible format to HCWs and Non-HCWs alike. This, in turn, will increase adherence to public health measures, reducing infections and ultimately saving lives.  

## Appendices

Appendix 1 

1.     How old are you (years)? (Single choice answer) 

·       Under 18 

·       18-39 

·       40-49 

·       50-59 

·       60-69 

·       70+ 

2.     Where are you currently residing in the UK? (Single choice answer) 

·       Greater London 

·       South East 

·       South West 

·       West Midlands 

·       North West 

·       North East 

·       Yorkshire and the Humber 

·       East Midlands 

·       East Anglia 

·       Scotland 

·       Wales 

·       Northern Ireland 

3.     Which of the following best describe your current occupation? (Single choice answer) 

·       Agriculture, Food and Natural Resources 

·       Architecture and Construction 

·       Arts, Audio/Video Technology and Communications 

·       Business Management and Administration 

·       Dentist 

·       Doctor 

·       Education and Training 

·       Finance 

·       Government and Public Administration 

·       Hospitality and Tourism 

·       Human Services 

·       Information Technology 

·       Law, Public Safety, Corrections and Security 

·       Manufacturing 

·       Marketing, Sales and Service 

·       Science, Technology, Engineering and Mathematics 

·       Nurse 

·       Allied Health Care Professional 

·       Medical or Nursing or Healthcare Student 

·       Student 

·       Transportation, Distribution and Logistics 

·       Unemployed 

·       Retired 

4.     Where do you get the majority of your information regarding Coronavirus? (Single choice answer) 

·       Facebook 

·       Twitter 

·       Group messaging services such as WhatsApp 

·       BBC News 

·       Daily Mail 

·       The Sun 

·       Metro 

·       Guardian 

·       Telegraph 

·       Research sites e.g. PubMed or Google Scholar 

·       World Health Organisation 

·       NHS England 

5.     Are you currently or have you previously self-isolated due to Coronavirus? (Single choice answer) 

·       Yes 

·       No 

6.     What, if any, steps are you taking to avoid transmission of Coronavirus? Please tick all that apply (multiple choice answer) 

·       None 

·       Avoid crowded public places 

·       Avoid going to work (working from home) 

·       Avoid raw meat consumption 

·       Avoid Chinese food consumption 

·       Buying increased amounts of cupboard food e.g. pasta, tinned goods 

·       Wear a face mask in public places 

·       Avoid contact of objects in public place e.g. door handles, elevator buttons 

·       Avoid contact of animals 

·       Improve personal hygiene e.g. washing hands more frequently, avoid touching face 

·       Buying increased amounts of medications e.g. paracetamol 

·       Buying increased amounts of hygiene essentials e.g. toilet paper, face tissues 

·       Buying increased amounts of cleaning essentials e.g. antiseptic wipes, hand soap 

7.     How long do you think the Coronavirus pandemic will affect your day to day life? (single choice answer) 

·       Less than 1 month 

·       1 - 2 Months 

·       3 - 4 Months 

·       4 - 6 Months 

·       6 - 12 Months 

·       Over 12 Months 

8.     How concerned are you about Coronavirus in regards to your own health? (Scale of 1 - 10 with 1 being ‘Not at all concerned’ and 10 being ‘Very concerned’)  

9.     How concerned are you about Coronavirus affecting your family's health? (Scale of 1 - 10 with 1 being ‘Not at all concerned’ and 10 being ‘Very concerned’) 

10.  How likely are you to avoid visiting elderly relatives? (Scale of 1 - 10 with 1 being ‘Not at all likely and 10 being ‘Very likely’) 

11.   How likely are you to avoid public places, including pubs, clubs and restaurants? (Scale of 1 - 10 with 1 being ‘Not at all likely and 10 being ‘Very likely’) 

12.  How concerned are you about Coronavirus impacting on your employment? (Scale of 1 - 10 with 1 being ‘Not at all concerned’ and 10 being ‘Very concerned’) 

13.  How do you feel the general public's reaction to Coronavirus is? (Scale of 1 - 10 with 1 being ‘Not concerned enough about Coronavirus’ and 10 being ‘Are over-reacting to Coronavirus’) 

14.  How do you feel the Government's reaction to Coronavirus is? (Scale of 1- 10 with 1 being ‘Not placing enough restrictions/not taking enough action’ and 10 being ‘Placing too many restrictions/over-reacting’) 

15.  How many Coronavirus positive patients do you think there are currently in the UK? (Single choice) 

·       The same amount as those officially tested positive 

·       2 x the amount as those officially tested positive 

·       5 x the amount as those officially tested positive 

·       10 x the amount as those officially tested positive 

·       100 x the amount as those officially tested positive 

·       1000 x the amount as those officially tested positive 

16.  Do you expect a public lock down similar to that in Italy, if so, how long for? (Single choice) 

·       I don't expect a public lock down 

·       1 - 2 weeks 

·       2 - 4 weeks 

·       1 - 2 months 

·       2 - 4 months 

·       4 - 12 months 

·       Greater than 12 months 

17.  How many UK Citizens do you think will die due to the Coronavirus Pandemic (Single choice) 

·       Less than 500 

·       500 - 1000 

·       1000 - 10,000 

·       10,000 - 100,000 

·       100,000 - 500,000 

·       More than 500,000 
